# Knowledge Discovery from Vibration Measurements

**DOI:** 10.1155/2014/917524

**Published:** 2014-01-16

**Authors:** Jun Deng, Jian Li, Daoyao Wang

**Affiliations:** ^1^School of Civil and Transportation Engineering, Guangdong University of Technology, Guangzhou 510006, China; ^2^Guangdong Provincial Academy of Building Research, Guangzhou 510500, China

## Abstract

The framework as well as the particular algorithms of pattern recognition process is widely adopted in structural health monitoring (SHM). However, as a part of the overall process of knowledge discovery from data bases (KDD), the results of pattern recognition are only changes and patterns of changes of data features. In this paper, based on the similarity between KDD and SHM and considering the particularity of SHM problems, a four-step framework of SHM is proposed which extends the final goal of SHM from detecting damages to extracting knowledge to facilitate decision making. The purposes and proper methods of each step of this framework are discussed. To demonstrate the proposed SHM framework, a specific SHM method which is composed by the second order structural parameter identification, statistical control chart analysis, and system reliability analysis is then presented. To examine the performance of this SHM method, real sensor data measured from a lab size steel bridge model structure are used. The developed four-step framework of SHM has the potential to clarify the process of SHM to facilitate the further development of SHM techniques.

## 1. Introduction

During the last two to three decades, many science in various areas have moved from a situation of a lack of (electronically) readable information into a situation of abundant data. The problem of extracting information from large masses of data became more and more important. Also knowledge became a very precious commodity for data application. Finding useful information or patterns in raw data is a rapidly growing area of research and application that builds on techniques and theories from many fields, including statistics, databases, pattern recognition and learning, data visualization, uncertainty modeling, optimization, and high performance computing. Knowledge discovery in data bases (KDD) concerns several research areas including data preprocessing, data modeling and feature extraction, pattern recognition as well as information visualization, and so forth [1]. The KDD process generally involves four steps: data preprocessing, transformation/feature extraction, data mining, and interpretation/evaluation [2]. The first three steps of the whole process are a pattern recognition problem whose results are generally formal patterns, such as clusters and sets of rules. In order to become knowledge or to be useful to human decision makers, they have to be interpreted with respect to the problem considered [1].

In the meanwhile, the structural health monitoring techniques are attracting more and more interests in civil engineering. The process of implementing a damage detection strategy based on sensor measurements (most generally, vibration measurements) for infrastructure is referred to as structural health monitoring (SHM) [3]. This definition clearly shows that SHM can be seen as a process to extract knowledge regarding structural working state from vibration measurements. Therefore, SHM problem has some similarities with KDD. In most recent years, some concepts of KDD especially the concept of statistical pattern recognition have been applied to SHM processes and several advanced techniques of pattern recognition can be found the applications in SHM applications.

In [3], the authors state “the SHM problem is fundamentally one of statistical pattern recognition.” They discussed SHM as a four-part process: (1) operational evaluation, (2) data acquisition, fusion, and cleansing, (3) feature extraction and information condensation, and (4) statistical model development for feature discrimination. In the meanwhile, the specific pattern recognition techniques, including neural network, support vector machines, statistical control chart analysis, and genetic algorithms have been used for structural damage assessment [3–8].

The successful applications of the pattern recognition concept as well as particular pattern recognition methods have enhanced the advance of SHM research. However, close comparison of KDD with SHM problem reveals the following three insufficiencies.

1. The importance of interpretation/evaluation step of KDD is omitted in the current SHM framework. As discussed, the pattern recognition is not a complete knowledge discovery process without interpretation/evaluation step since its results are only information regarding the pattern of the data. If the SHM problems are analyzed with only pattern recognition techniques, the final results are the information regarding system changes and the patterns of these changes. However, SHM is not a pure damage detection problem. Information regarding the working state of structural system should be provided to facilitate the decision making about if/what actions should be taken to repair the structure.
2. Although SHM can be seen as a KDD problem, SHM problems have their own particularities. The knowledge to be extracted in KDD is the artificial “intelligence” such as implication or correlation between data. In SHM, the knowledge we are heading to is not correlation between data but the state of the structure. Therefore, the importance of correlating data with structure states should be emphasized in the SHM framework which is not an issue in KDD based on purely statistical analysis.
3. Some pattern recognition methods have their limitations to be applied on SHM. Some pattern recognition methods are based on the clearg definition of patterns. However, in SHM, data from damaged systems are generally not available. It is not possible to predefine the patterns of damages without sufficient measurements. These challenges are supplemented by many practical issues associated with making accurate and repeatable dynamic response measurements at a limited number of locations on complex structures often operating in adverse environments.

To address these issues, this paper starts from the discussion of SHM problems on the context of the knowledge discovery. Adopting the KDD concept, in the meanwhile, considering the particularities of SHM problems, we propose a four-step framework of SHM: data preprocessing, feature extraction, feature analysis, and system evaluation. The SHM framework discussed in this paper assumes the response measurements are available. The data transmission, management, and retrieval in SHM system are out of the range of this paper. We shall then present a specific SHM method which is composed by second-order structural parameter identification as feature extraction method, statistical control chart analysis of identified stiffness for feature analysis step, and system reliability analysis based on identified stiffness for structural system evaluation. To demonstrate the performance of the proposed SHM method, real sensor data measured from a lab size steel bridge model structure were used in this study. Compared with the specific method proposed here, the developed four-step process of SHM will provide a more general framework for SHM applications in practices.

## 2. Knowledge of SHM

Knowledge is defined as the fact or condition of knowing something. To understand what is the specific “knowledge” that the SHM method should provide, it has to be interpreted with respect to the goals of SHM. SHM is aimed at facilitating the decision maker on the structural maintenance and repair. Usually, the goal of SHM methods can be categorized into four stages: (1) detecting the existence of damage, (2) locating the damage, (3) determining the severity of the damage, and (4) evaluating the remaining capacity of the structure. Therefore, the output of SHM methods should be the facts of structural damages and the damaged structure system.

Another important requirement of “knowledge” is that it should be directly readable and usable. Since the final knowledge users are the decision makers in SHM, in other words, the results of SHM methods have to be translated into the language of the decision makers by directly related to if/what actions should be taken.

In current practices, most SHM methods involve two steps. In first step, the data features are extracted from vibration measurements. Various features have been applied for structural damage detection including modal properties (modal frequencies, modal shapes, etc.) and various mathematical model parameters. In the second step, the features changes are related to structural system changes by direct comparison with feature baselines or more advanced statistical analysis including feature classification. Therefore, the results of these methods are information about possible structural damages. The results cannot reach the four-stage goals of SHM since no information regarding the damaged structure is provided. In the meanwhile, the results cannot directly facilitate decision making since the detected damages are not directly related to if/what actions should be taken. These methods simplify SHM into pure damage detection problems; thus, they cannot provide comprehensive knowledge for decision makers. It is necessary to adjust the goal of SHM from detecting damage to extracting real knowledge necessary for making decisions.

## 3. A Four-Step Process of SHM

**Figure 1:**
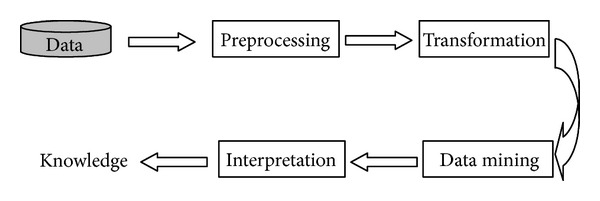
The steps of the KDD process.

KDD in computer science is the process of automatically searching large volumes of data for association rules based on purely statistical analysis such as information retrieval, machine learning, and pattern recognition. The process of knowledge discovery in data bases (KDD) normally contains a number of steps, as shown in Figure 1 [1, 2]. The intended goals of individual steps are explained as follows.

1. Preprocessing: this step is intended to select, clean, and correct data to facilitate further KDD analysis. Real data contains noise and has outliers and irrelevant information. Data is missing and might be wrong. Therefore, selecting, cleansing, and correcting data before knowledge discovery very often not only facilitate KDD but make it possible.
2. Transformation/feature extraction: in KDD, this step is the first step of dimensionality reduction of large-scale data bases. Features which are relevant for the goal of the KDD are determined. The space that the features span has a lower dimension than the data space. Feature analysis makes the following mining process most efficient without losing any important information if the features are most relevant to knowledge to be extracted.
3. Data mining: this is the actual pattern recognition step. It extracts patterns represented by data based on feature analysis. The most common types of patterns in data are relationships between objects, temporal sequences, spatial patterns, sets of similar objects, mathematical laws, and so forth, [9].
4. Interpretation/evaluation: the results of data mining are generally formal patterns, such as clusters, sets of rules, partitions according to multidimensional attributes. In order to become knowledge or to be useful to human decision makers, they have to be interpreted with respect to the problem considered. In other words, the results have to be translated into the language of the observer.

Aimed at extracting directly usable knowledge from abundant data to facilitate decision making, SHM problem has lots of similarity with KDD. The detailed description of KDD process helps us to review and classify current SHM methods and to further develop a general framework for SHM methods. Compared with KDD process, the current two-step SHM methods mentioned above are only focused on feature extraction and feature analysis steps. In the meanwhile, feature analysis very often only contains directly comparison between features with feature baselines. Therefore, inspired by the KDD process, it could be expected that the current SHM methods can be further improved from the following aspects.

1. Data preprocessing: data preprocessing has been incorporated in the SHM methods as a critical step in the SHM framework proposed by Sohn et al. [3]. The importance of data preprocessing has been realized in some extend. Staszewski [10] examines various preprocessing techniques that enhance feature extraction and selection. The authors believe that incorporating data preprocessing step in the SHM method can improve the efficiency and accuracy of SHM.
2. Detailed feature analysis: in current SHM methods, feature extractions are well studied. Various features including both model-based and nonmodel-based features are proposed and examined for structural damage detection. In contrast, the feature analysis method is relative simple. Features representing local properties of dataset with limited time window might be affected by local properties of excitation and noises, environmental changes, and even unpredicted factors. Advanced statistical analysis can filter out the irrelevant factors, and hence, improve the accuracy of extracted pattern. The advanced statistical pattern recognition methods are efficient tools to be applied in this step.
3. Structural system evaluation: the final results of current SHM methods generally are information about possible damages. The authors believe that the SHM is not exactly equal to damage detection. Information regarding damages is not all knowledge which can be extracted from data to facilitate the decision makers. Structural system evaluation based on the detected structural changes can translate the information from only damage related into system related which might be directly related to if/what actions should be taken.

On the other hand, although SHM can be seen as a KDD problem, SHM problems have their own particularities. As discussed, the knowledge to be extracted in KDD is the artificial “intelligence” such as implication or correlation between data. In SHM, the knowledge we are heading to is the state of the structure. KDD is trying to find out the patterns of how the displacement and acceleration are related. SHM is trying to figure out the “health” state of the structure from which the data are measured. This difference could be considered from two ways: first, the feature extracted from original data in SHM should represent not only the data properties but also the structural properties. For example, the modal frequencies extracted from vibration data is better feature for SHM applications than response amplitude since it is closely related to structural properties. Second, the classified pattern should be further interpreted to correlate individual pattern with structural states. The difference of final knowledge to be extracted between KDD and SHM determines the difference on their analysis methods. Purely statistical analysis methods of KDD cannot completely solve SHM problems. Structural dynamics and structural analysis techniques are necessary to be incorporated. Therefore, based on the similarities and differences between KDD and SHM, taking advantages from both KDD and SHM techniques, the SHM process can be divided into following four steps.

1. Data preprocessing: this step is aimed at enhancing the efficiency and accuracy of the following analysis. For the efficiency consideration, selecting data which detailed analysis are possible and necessary to be performed on can release the burden of analysis. In the meanwhile, selecting data which better satisfies the assumptions or requirements of the detailed analysis method can improve the accuracy of the analysis.
2. Feature extraction: for SHM applications, the extracted feature should satisfy two requirements: same as in KDD, the feature should reduce the dimension of original data. And, the feature should be sensitive to the structural damage. The widely studied structural system identification method could be seen as a feature extraction method specially fitting the SHM applications. The system identification methods extract system properties such as modal frequencies, modal shapes, and stiffness, from set of vibration measurements. These features are directly related to the physical properties of structures. Hence, analyzing these features with reduced data dimension could efficiently provide information about structure system.
3. Feature analysis: damage in SHM can be defined as changes introduced into a system that adversely affects its current or future performance [3]. In the meanwhile, features present the properties of the system contained in the datasets collected at the particular moment. Therefore, damage is not meaningful without a comparison between two different states of the system [3]. On the other hand, the features might be affected by various factors such as local properties of dataset and environmental changes. Statistical pattern recognition methods are beneficial not only to confidently reveal the changes but also classify the change patterns which are related to details of the changes and the reasons caused these changes.
4. System evaluation: as discussed, the final goal of SHM is to provide information about structural safety to facilitate decision making. The pattern recognition procedure consisted by above mentioned three steps provides information about system changes. Damages are possible factors to affect system safety, but not real state of the structural “health.” Respecting the SHM problem, these damage-related pieces of information should be further translated into knowledge about structural working state to help decision makers understand the damaged structural system and taking necessary actions. System reliability analysis and structural performance prediction based on the updated system properties are possible methods to evaluate the damaged system.

## 4. SHM Based on Identified Structural Stiffness

To demonstrate the proposed four-step framework of SHM, in this section, a specific SHM method is presented which is composed by second-order structural parameter identification as feature extraction method, statistical control chart analysis of identified stiffness for feature analysis step, and system reliability analysis based on identified stiffness for structural system evaluation. Details of these methods are presented as follows.

### 4.1. Data Preprocessing

As discussed, data preprocessing is aimed at improving the accuracy and efficiency of further analysis. Since a PEM-based second-order structural parameter identification method is applied in the feature extraction step, from accuracy consideration, the dataset to be used should satisfy the assumptions of the identification method. In the identification method, the structure will be firstly idealized into a simple dynamic model which can only model the global modes of the system dynamic properties. The structural stiffness, mass, and damping ratios corresponding to the idealized model will then be directly identified from response measurements. Therefore, the dataset to be identified should contain enough information corresponding to first several modes of the structure. The response data dominated by higher modes which cannot be modeled by the idealized simple model is not suitable for this method. Hence, in the data preprocessing step, response data should be selected based on this criterion. In the meanwhile, in a long-term monitoring system, response data are accumulated with time. It is not efficient, even not possible to retrieve and analysis all data. A PCA-based data management and retrieval method are proposed by authors to select dataset corresponding to system changes [11]. This method can be also seen as a data preprocessing method to select data for further analysis based on efficiency consideration.

### 4.2. Feature Extraction

In the proposed SHM method, a time-domain structural system identification method is used to extract structural stiffness as features from vibration response measurements in the feature extraction step. This method can identify second-order structural parameters even under unknown excitation conditions. The structural identification method involves the use of PEM method to extract second-order structural parameters including mass, stiffness and damping ratios directly from measured vibration data.

The proposed second-order structural identification method starts with idealizing the original structural into a simplified dynamic model and, then, expands the state space model of the simple model into linear models which have parameters as functions of second-order structural parameters. Then, PEM method is used to estimate the unknown second-order model parameters.

The final mathematic models to be identified are as follows.

When input excitation is available,

(1) $$\begin{array}{c} \overset{2N}{\underset{i=0}{\sum }}\alpha_{i}\left(\theta \right)\cdot y_{k-i}=\overset{2N}{\underset{i=0}{\sum }}\beta_{i}\left(\theta \right)\cdot u_{k-i}+\overset{2N}{\underset{i=0}{\sum }}\gamma_{i}\left(\theta \right)\cdot e_{k-i}, \end{array}$$

output only situation:

(2) $$\begin{array}{c} \overset{2N}{\underset{i=0}{\sum }}\alpha_{i}\left(\theta \right)\cdot y_{k-i}=\overset{2N}{\underset{i=0}{\sum }}\gamma_{i}\left(\theta \right)\cdot e_{k-i}. \end{array}$$

The unknown second-order structural parameters to be identified are denoted as vector *θ*. To identify model described in (1), both excitation force and structural response measurements are needed. The whole unknown second order structural parameters including mass, stiffness, and damping ratios can be identified simultaneously. The model described in (2) is based on the Gaussian distributed ambient excitation assumption. Only dynamic responses are necessary to identify this model. Because of lack of information about excitation, only part of second-order parameters (mass or stiffness) can be identified from this model. Based on these two models, a two-stage SHM application is proposed: the first stage entails the identification of all second-order structural parameters of linear structures from controlled vibration tests with known input. The second stage involves output-only structural identification which can be applied to ambient vibration applications with unknown inputs and limited number of output measurements. In the second stage, the structural masses identified from stage one are assumed to be unchanged and only stiffness parameters are identified. Stiffness parameters are then the final features to be further analyzed. Damage is located and quantified through the changes in the identified stiffness coefficients and system reliability will be further performed based on the identified stiffness parameters.

Since the identification method directly extracts second-order structural parameters as features which are mathematical descriptions of structural physical properties, using the identified results as features can present comprehensive knowledge regarding structural damage locations, damage severity, and remaining capacity of structures. Details about this identification method are presented in [12].

### 4.3. Feature Analysis

The statistical control chart analysis of the identified stiffness is adopted in this step to extract information about possible structural damages. As mentioned in the previous sections, the features used in this method are structural stiffness which already closely correlated with structural properties. The feature changes themselves indicate comprehensive information about damage. It is not necessary to define complicated pattern corresponding to damage locations and severities. Therefore, the feature analysis method used in this method is relatively simple. The only concern in this stage is then confidently classifying changed stiffness coefficient. The system identification methods generally can only be applied on a set of data with limited time window. Data from each set may have local properties because of the limited duration of time window. The features including aforementioned identified stiffness parameters might fluctuate within a particular range among the datasets even though they are collected from the same structure. Therefore, it is necessary to define the confident range of the feature which can classify the original system from systems with changes. The statistical control chart analysis method is used in the proposed SHM method to analyze these features from statistical point of view. To plot statistical control chart, the confident range of features must be identified from history data. Mathematically, to define the feature range with *α*% confidence, the upper and lower control limits (denoted as UCL and LCL resp.) can be written as

(3) $$\begin{array}{c} \text{UCL}=\text{invnorm}\left(1-\frac{\alpha }{2}\%,\bar{F},S_{F}\right),\\ \text{LCL}=\text{invnorm}\left(\frac{\alpha }{2}\%,\bar{F},S_{F}\right), \end{array}$$

where invnorm denotes the inverse function of the probability density function of normal distribution and $\bar{F}$ and *S*_*F*_ are the mean value and standard deviation of the identified stiffness in this application. The above equations are based on the Normal distribution assumption for the extracted features from the history measurements. However, it has been shown that the control limits based on the Normal distribution assumption can often be successfully used unless the population is extremely nonnormal [13, 14]. Note that *α*% is the confidence value that classifies the system similar to the original system from which the history measurements were collected. Overly defined *α*% will cause higher likelihood for misclassifying systems with changes. Since the structural damages are always reflected as stiffness reduction, only lower control limits of identified stiffness are meaningful for damage classification.

The final results of this step are then the location and magnitude of the changed stiffness. Hence, the analysis procedure is performed on the statistical sense. This step also updates the statistical properties of the changed stiffness coefficient which could be used for structural reliability analysis in the next step. Although it is not presented in this paper, the updated stiffness parameters can also be applied to update the structural models and more knowledge regarding structural current state could be acquired through the analysis of the updated models.

### 4.4. System Reliability Evaluation

In most of current SHM methods, the analysis procedure stops at the detection of damages. The system evaluation after possible damages are detected is generally omitted. However, to make final decisions from information about damages, the further analysis is still necessary. The decision makers should be informed at least how significant the overall system is affected by these damages. In the proposed SHM method, we use the identified structural stiffness parameters to update the system reliability to indicate the severity of damage effects on system performance and the urgency of that action should be taken.

A reliable system is defined as one that is capable of operating without failure during a specified period in a specified environment. Failure is defined as the state in which the resistance *R* could not satisfy the demand *D* of the system. Probability of failure is then generally used to indicate the reliability of the system. In real applications, the demand of the system might be an assembly of different requests. The system reliability analysis is then a complicated statistical analysis process depended on a lot of information.

In this paper, we focus on introducing the framework of SHM using KDD concept especially on emphasizing the importance of system evaluation. System reliability analysis is proposed as one of possible methods to translate the information of damages into the knowledge regarding structural systems which can be directly used by human decision makers. In stead of studying specific system reliability method, we adopt simple reliability analysis concept to demonstrate our ideas.

Since the feature used in this method is structural stiffness coefficients along a particular direction and the loading conditions of structure is generally predefined, the structural demand we defined for the reliable system is simply the request regarding the response displacement of the structural system under regular loads. In particular, the demand used in the demonstrated reliability analysis is *D* = the displacement of the center point of the structure under predefined static load should be smaller than 1/20 of the span of the structure.

The real displacement responses of the structure under static load can be calculated from predefined load and identified stiffness as

(4) $$\begin{array}{c} R=K^{-1}\cdot F, \end{array}$$

where *F* is predefined static load of the structure. *K* is identified stiffness matrix. The structural resistance is then the displacement of the center point of the structure which is the element of *R* at center degree of freedom: *R*_*c*_. In this study, both predefined static load and identified stiffness are random variables. Therefore, real structural resistance *R*_*c*_ is also random variable.

The overall reliability of the system is then indicated by the probability of system failure:

(5) $$\begin{array}{c} P\left(R_{c}-D\geq 0\right) \end{array}$$

which will be calculated by Mento Carlo simulation based on the statistical properties of the updated stiffness coefficients and predefined static load.

## 5. Experimental Study

### 5.1. A Model Steel Bridge Structure

To demonstrate the concept and evaluate the performance of the SHM method described above, an experimental study is performed on a model steel bridge structure. The details of this model structure as well as the dynamic tests are given as follows.

**Figure 2:**
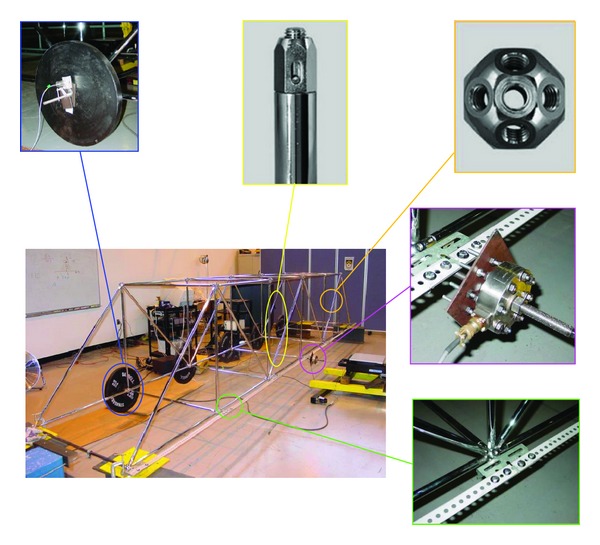
Configuration of the model steel bridge structure.

The structure being monitored is a model steel bridge structure. The steel bridge structure has a total of 24 nodes, 32 short rods with a 1 m length and 12 long rods with 1.414 m length assembled together using steel tubes, and nodes of M12 system from the MeroForm Systems.  Figure 2 shows the configuration of the steel bridge structure and its members and connections. The size of the assembled structure is 1 m × 1 m × 6 m (width, height, and length). The four end nodes in the lower plane of the steel bridge structure are restrained in translation direction at the supports. Five steel blocks with 45 lb (20.4 Kg) each are attached to the five lower nodes along one side of the lower plane to simulate structural mass. Additional slotted steel stripes are bolted to all seven nodes along the other side of the lower plane to provide additional lateral moment resisting stiffness in the transverse direction of the steel bridge structure. The reason for doing this is to simulate structure damages by loosening the connections of these steel stripes in subsequent tests.

**Figure 3:**
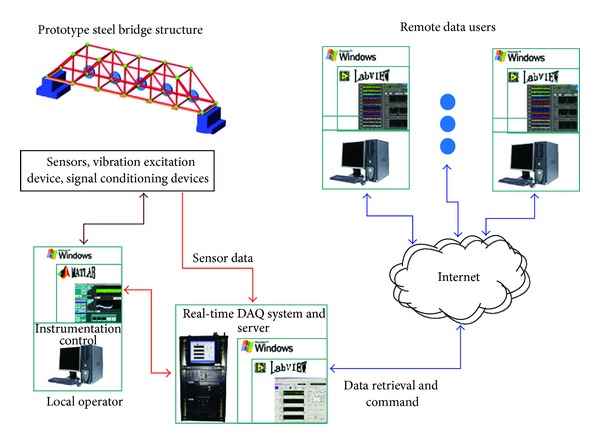
Configuration of the hardware system for experimental study.

Since the proposed SHM method is a vibration-based method, the major parts of the experimental hardware are related to vibration excitation and vibration response collection.  Figure 3 shows the configuration of this experimental hardware configuration. A long stroke shaker with a 6” peak-to-peak stroke and 100 lbs maximum output force from APS Dynamics, Inc., was used to excite the steel bridge structure at its middle node located in the lower plane. Five piezoelectric accelerometers (Model 393B04 from PCB Piezotronics, Inc.) were used to measure the acceleration responses of the steel bridge structure during the entire vibration test. The accelerometers were attached to the nodes along one side of the lower plane of the steel bridge structure. Only five accelerometers were used to measure the responses from a limited number of the structural nodes on the model steel bridge, which is aimed to examine the performance of the proposed SHM method using limited measurements—a situation often encountered in real civil engineering structures. The RTMS-2001 real-time data acquisition system from Digitexx Data Systems Inc. was used for force and acceleration data recording and broadcasting the sensor data to Internet for remote data access.

Since the system identification method has to be performed on a set of data, a time window had to be adopted to divide the continuously measured data into individual datasets. Each measurement set is denoted as one test in this study. One test thus defined here includes 1,024 data samples from each of the five accelerometers and therefore the entire dataset contains a total of 5,120 data samples. Since the sampling rate used by the data acquisition system was 200 Hz, the time window for dividing the measurements was therefore equal to 5.12 seconds.

### 5.2. Test Procedure

Before applying the proposed SHM method for knowledge discovery, initial parameters for the individual methods need to be determined using test data measured from the original structural system, which serve as a baseline for future analysis. The parameters that need to be set up from pretest measurements include the following.

- Mass and damping parameters for the steel bridge structure: as mentioned in above sections, the adopted second structural system identification method is a two-stage procedure. In the second stage which involves output-only identification, mass, and damping parameters shall become available from the first stage and are assumed to be unchanged. Therefore, a well-controlled modal testing must be conducted to identify the system mass and damping ratios in the first stage.
- Control limits of identified stiffness parameters: the identified stiffness parameters will be affected by the local properties of sensor data due to limited duration of the time window. Therefore, regressed control limits from a statistical analysis of pretest data will be used to enhance the accuracy of feature analysis.
- Statistical properties of stiffness parameters and the system reliability of original structure: these parameters are critical for the system reliability analysis of original structure.

To identify the abovementioned parameters beforehand, the whole test procedure for the steel bridge structure includes the following three steps.

**Step 1**

Well-controlled modal testing to identify the mass and damping ratios of the steel bridge structure.

**Step 2**

Output-only tests on the original bridge structure to identify the parameters as discussed above.

**Step 3**

Real application of SHM method to examine its performance in different damage scenarios with various system changes.

### 5.3. Well-Controlled Modal Testing

For output-only monitoring of stiffness, the mass and damping ratios of the steel bridge structure need to be identified beforehand in Step 1  by a well-controlled model testing. A well-controlled model testing is defined as (i) measurements of the input excitation are available and must be force measurements; (ii) the colocation requirement needs to be satisfied; colocation means that there exists at least one DOF with both a sensor and an actuator.

**Figure 4:**
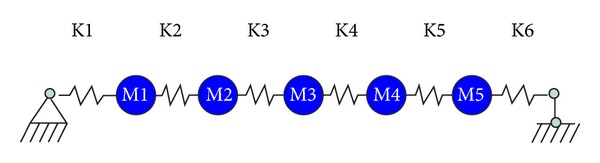
Idealized lumped-mass model for the steel bridge structure.

In this stage, the original steel bridge structure was excited at the middle node and the input dynamic force was measured using the load cell. The acceleration responses of the bridge at five nodes were measured using five accelerometers. A total of six tests were conducted with 1,024 data samples from each sensor. The measured dataset was used to identify the second-order structural parameters of the bridge structure using the method described above. Therefore, six sets of second order structural parameters were identified from these measurements. The average values of mass and damping ratios of these six groups are taken as the parameters which will be used in the subsequent online monitoring stage. To identify the second order structural parameters, the original steel bridge structure is idealized into a 5-DOF lumped-mass model shown in Figure 4. A total of 16 unknown parameters thus need to be identified as second order structural parameters including 5 masses, 5 damping ratios, and 6 stiffness parameters. All 16 unknown parameters can be identified simultaneously from the measurements in each test.

**Figure 5:**
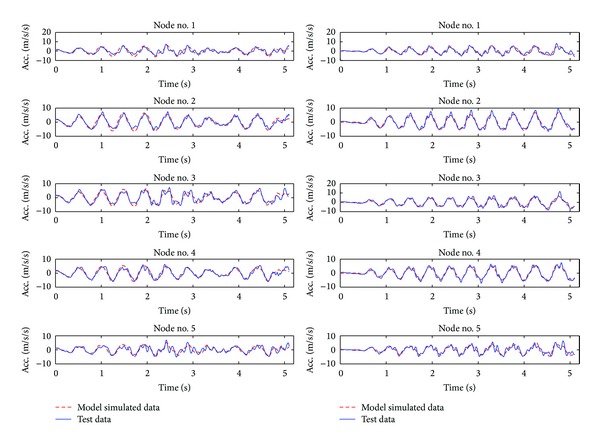
Comparison of two of total six test results (blue solid) and numerical simulation (red dashed) results using identified model.

Table 1 lists the identification results for the original undamaged bridge structure from the measurements in these six tests.  Figure 5 shows the comparison of the simulated responses using the identified model and two sets of real measurements in each test. It is seen that the idealized lumped-mass model with identified second-order parameters can model the original structure reasonably well. The mismatch over higher frequency contents in these measurements is believed to be caused by higher order vibration modes which go beyond the highest modes of this lumped-mass model.

### 5.4. Pretests for Parameters Identification

In this step, a total of 151 tests each with duration of 5.12 seconds were conducted on the original bridge structure to identify the abovementioned parameters for further analysis. In these tests, acceleration responses of the bridge structure were the only quantity to be measured using a total of 5 accelerometers.

The first parameter to be identified in this step is the control limits of the identified stiffness coefficients. The variation of the stiffness values identified from each test dataset was observed in the pretest identification results, which was attributed to the local statistical properties of the input excitation to the system. This shows the importance of the control chart analysis in the feature analysis stage. The objective of setting up the control limits for identified stiffness parameters is to confidently classify damaged structures from undamaged structures based on the identified stiffness values. An identified stiffness value which falls within the confident range of the undamaged structure implies that the likelihood for the structure to be damaged is low. Since structural damages in this study are characterized by stiffness reduction, only lower limit needs to be determined for our application.

**Figure 6:**
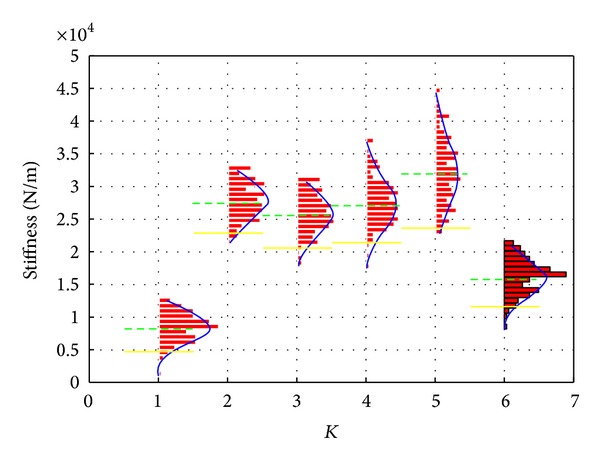
Control chart for identified stiffness calculated from pretest measurements.

Another approach to analysis damage based on identified stiffness control chart is to compare the mean value of identified stiffness results from several test measurements with the pretest values of corresponding stiffness. Even if a single identified value cannot be confidently classified as being damaged using the control limits because of the variation, mean value of several tests results can enhance the confidence of classification by comparing mean values. Therefore, constructing the control limits for stiffness has two subtasks which include the calculation of lower control limit and calculation of mean values for the stiffness identified from the 151 pretest measurements.  Figure 6 is the final control chart regressed from pretests measurements which can be used for feature analysis in subsequent applications.

Other important parameters to be regressed in this stage are the statistical distribution of the stiffness parameters and the reliability analysis of original structure. In this test, since only limited number of tests were conducted which are not sufficient for performing accurate Mante Carlo Simulation, the statistical properties of the identified stiffness parameters are calculated and the normal distributed random variables of these parameters are then generated based on their statistical properties. In particular, we first calculate the mean and standard deviations of six stiffness parameters from 151 sets of identified stiffness parameters. Then, 10000 samples of stiffness parameters are generated based on the assumption that the stiffness parameters are normal distributed random variables with determinate mean and standard deviations. In real applications of SHM practices, especially in on-line monitoring system where data are accumulating with time, large number of sets of stiffness might be identified from available data. It is not necessary to assume the distribution of stiffness parameters. The reliability analysis could be performed more accurately.

As mentioned before, the static load of the structure is assumed to be available in the reliability analysis which is assumed to be normal distributed random variables with mean value and standard deviation equal to 2500 N, 250 N, respectively. The demand to be satisfied is only the midpoint displacement. The probability of system failure could be calculated by Monte Carlo Simulation with sample size 10000.

**Figure 7:**
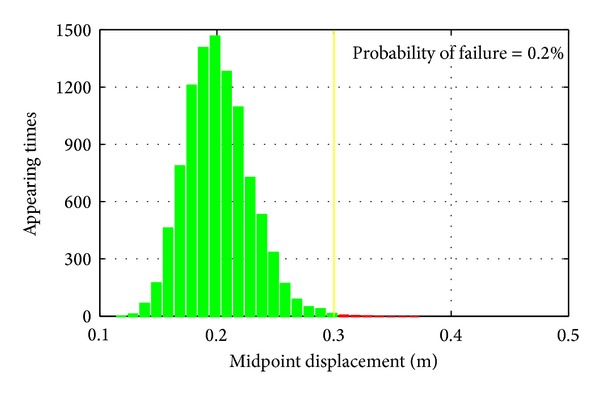
Reliability analysis of original structure.

Figure 7 shows the histogram of the calculated midpoint displacement by Monte Carlo Simulation based on the identified stiffness statistical properties. Compared with defined demand of midpoint displacement as shown in solid line in Figure 7, the probability of failure of original structure is 0.2% which is very small.

### 5.5. Structural Health Monitoring

After setting the aforementioned parameters from the pretest measurements, the proposed method is ready for structural health monitoring applications. In this experimental study, various damage scenarios which were simulated in the steel bridge structure were conducted to evaluate the performance of the proposed integrated SHM system. Limited by the paper length, only 4 damage scenarios are presented herein which are corresponding to single damage case, changed damage locations, multiple damage case, and retrofitted structure case.

- Damage scenario no. 1: the steel stripe connection at K1 location (see Figure 4) was completely disattached from the primary structure by loosening the bolts.
- Damage scenario no. 2: the steel stripe connection at K2 location was completely disattached from the primary structure to simulate changed damage locations.
- Damage scenario no. 3: the steel stripes at K3, K4, and K5 locations were simultaneously disattached from the primary structure to simulate the multiple damage case.
- Damage scenario no. 4: all steel stripes were connected back to the primary structure to simulate the retrofitted structure case.

In this study, damages to the steel bridge structure were simulated by loosening the connections between the steel stripes and the primary structure as described above. Stiffness reduction is introduced to the particular locations where the stripe connection was disattached from the primary structure. Due to the configuration of the steel bridge structure, loosening the steel stripe connection at one location would also influence other steel stripe connections, particularly those in neighboring spans. This is also verified in the subsequent tests. For each damage scenario test, a total of 15,360 data samples were continually measured from each accelerometer attached to the nodes. These measurements can be divided into 15 tests (15,360 = 15 × 1,024) using a predefined time window. From hereforth, the results will be presented in terms of test numbers instead of data sample numbers. The input excitations in all these damage scenarios were Gaussian white noise to simulate ambient excitation in real structures. The discussions of individual damage scenario are presented as follows.

**Figure 8:**
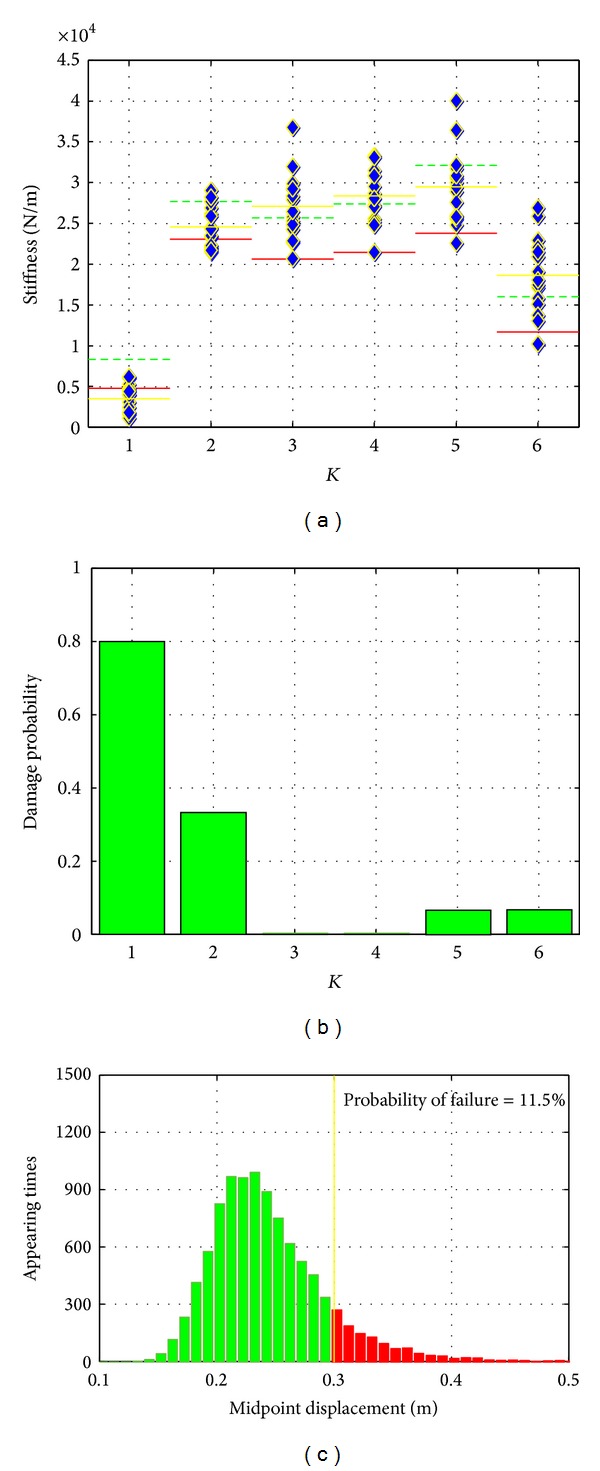
Results of damage scenario no. 1.

*Damage Scenario No. 1*.  Figure 8 plots the results from monitoring the steel bridge structure with damage scenario no. 1. The first step for data analysis in the proposed SHM method is data preprocessing. As mentioned in previous section, the data will be selected based on the criteria of fitting the system identification method. In particular, the acceleration responses should contain enough information in the frequency band around the first few modal frequencies of the original structure. In this experimental study, we excited the structure with Gaussian distributed white noise with frequency bond 0–5 Hz which is close to the frequency range of the first five modes of the structure. The first five modal frequencies of the model steel bridge structure are 1.64 Hz, 3.96 Hz, 6.24 Hz, 9.00 Hz, and 11.09 Hz, respectively. Therefore, the responses are dominated by first five global modes of the structure. Higher mode responses in the collected data are less dominated. The collected data were directly applied for system identification and further analysis. In practice, since the excitation inputs are not controllable, the response data should be selected and preprocessed to satisfy this requirement.

In the second step of the SHM method, the downloaded data will be analyzed using the second-order structural system identification method as described in previous section. This system identification method directly identifies structural stiffness from ambient vibration data without the need for excitation input measurement. The mass and damping ratio identified from well-controlled modal testing were used for the stiffness identification.

After stiffness parameters were identified from data, the statistical control chart analysis is then performed on these identified features to extract information regarding possible damages. The statistical analysis of the identified stiffness during the pretest stage provides the confidence limits for the structural stiffness. An identified stiffness value lower than the confidence limit will be classified as damage with 90% confidence. In the meanwhile, comparing the mean value of the identified stiffness between the original structure (presented as dashed line) and of damaged structure (presented as yellow solid line) in Figure 8(a) also provides an alternative way of damage detection as mentioned earlier. Figure 10(a) shows the identified stiffness for the six spans of the steel bridge structure from all 15 test datasets in this damage scenario.  Figure 10(b) shows the normalized occurrence frequency for the individual stiffness that is confidently classified as being damaged in these 15 tests which is indicated by identified value over the predefined limit. Combining the results given in these two figures shows that identified K1 was outside the limits 12 times out of a total 15 and the mean value of these 15 identified stiffness values was much smaller than the mean value corresponding to the original structure as shown by the dashed line. Therefore, we can confidently conclude that the K1 was damaged. For K2, although only 5 identified results from these 15 tests are confidently classified as being damaged, other identified values within the limits also show certain level of reduction compared with the mean value of the pretest results. Certain occurrence frequencies of confidently classified as being damaged and the apparent difference between the average stiffness value of these 15 tests and that of the 151 pretests indicate that K2 had some smaller level damage. The reduction of the mean values of identified stiffness shows that the level of damage in K2 was less severe than that in K1. Other stiffness parameters such as K4 cannot be classified as being damaged since no or only one identified result out of a total of 15 identified stiffness values went outside the predefined limits and yet no significant change was observed in average stiffness values. Therefore, the finally acquired knowledge is that K1 was severely damaged and K2 was slightly damaged. Since the identified feature was the structural stiffness, the remaining capacity of the damaged structure may be predicted based on these identified results.

From previous steps, the damage locations as well as the quantity of stiffness reductions are detected. However, the system safety, in other words, how the damage affects the structural system, is still not clear. The structural system analysis is then necessary to be performed based on the updated system properties to further interpret the information of damages. The selection of particular method for system analysis based on information provided from first few steps should consider both the decision maker requirements and the reality of if the information extracted from first step is sufficient to support this method. In the proposed SHM method, the simple reliability analysis procedure described above is adopted in this step to demonstrate the SHM framework as well as the importance of this step. More advanced method could be developed in further study.

The statistical properties of the identified stiffness parameters acquired from first few steps were used to update the reliability analysis described above. Based on the updated statistical properties of the identified stiffness, 10000 samples of variables corresponding to six stiffness parameters are regenerated based on normal distribution assumption. Monte Carlo Simulation is then performed using these samples.  Figure 8(c) plots the histogram of calculated midpoint displacement of damaged structure. As shown in this figure, the probability of structural failure is increased to over 11% which indicates that the damages cause high risks of structural failure.

Through the whole analysis procedure, the knowledge provided to decision makers is then the structure is damaged at K1 and K2 locations. The damaged structure is under high risks of failure. Urgent actions should be taken to increase the stiffness K1 about 5000 N/m and the stiffness K2 about 3000 N/m. Clearly, these results could strongly support the decisions about structural repair.

**Figure 9:**
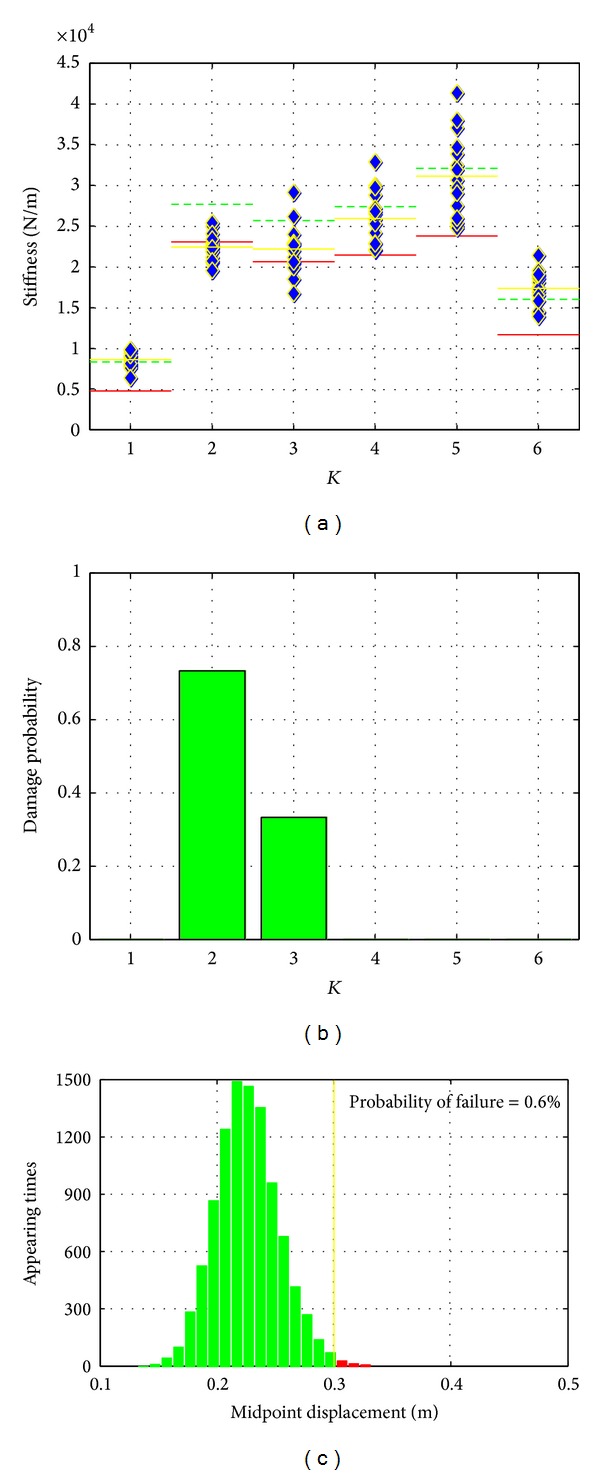
Results of damage scenario no. 2.

*Damage Scenario No. 2*. In this case, damage was simulated by loosening the connection of the steel stripe at K2 to validate the SHM method for different damage locations. Similar analysis procedure of the SHM method to that used in damage scenario no. 1 was applied and results were presented in Figure 9.

Output-only system identification was performed on the 15 tests data to determine the values of stiffness parameters of the steel bridge structure. The control chart analysis was carried out to extract the damage-related knowledge from the identified stiffness values. Similar to the analysis presented in damage scenario no. 1, the stiffness reduction of K2 and its effects on K3 was successfully identified. Compared with the mean value of K2 before and after the damage, about 15% reduction was observed for the damage introduced by loosening the K2 connection. The results shown here verified the effectiveness of the proposed integrated SHM system for damage detection.

The reliability analysis of this damage scenario is presented in Figure 9(c). Compared with damage scenario no. 1, the almost same stiffness reduction quantity at different locations caused significantly different effects on structure system. In this damage case, the probability of the structure failure is only slightly increased. Therefore, the structural repair is not as urgent as damage scenario 1.

The comparison of damage scenarios no. 1 and no. 2 verified the importance of system analysis step. Damage detection itself could provide the information regarding system. The same quantity of damages might cause different effects on system. Hence, information should be provided to facilitate the decision makers making correct decisions.

**Figure 10:**
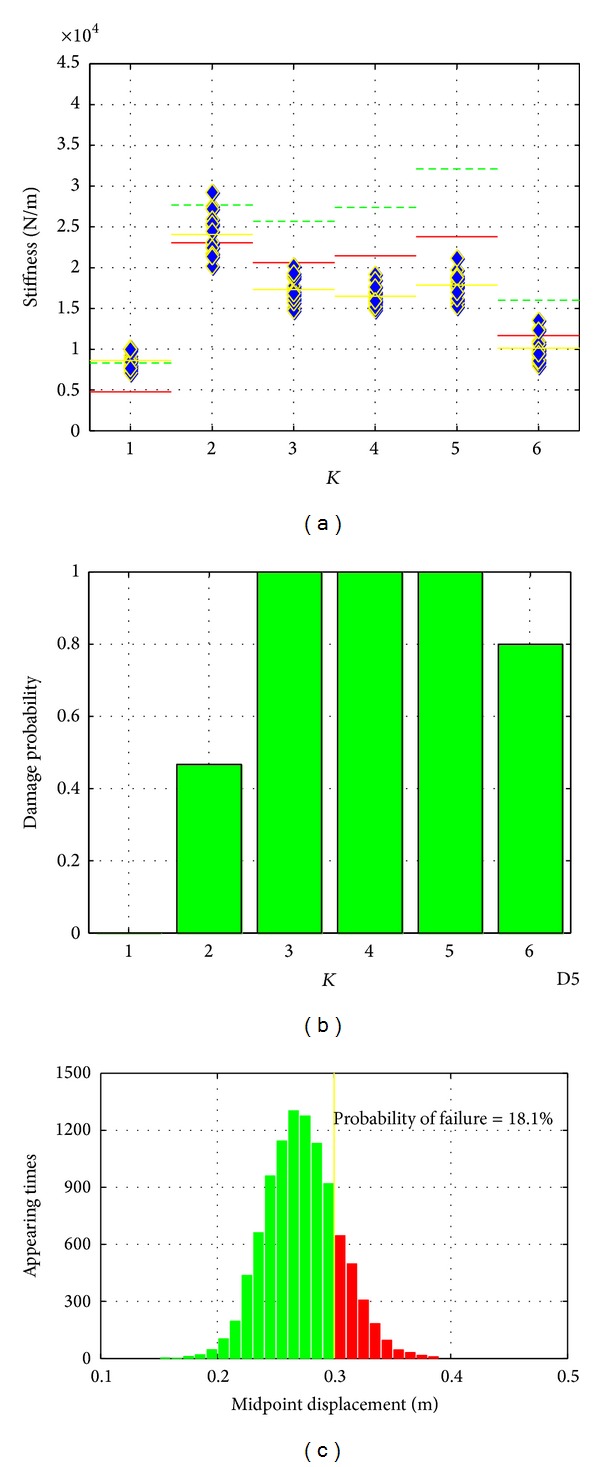
Results of damage scenario no. 3.

*Damage Scenario No. 3*. This case is presented to examine the performance of the proposed SHM method under the multiple damage situations. The steel stripes at K3, K4, and K5 locations were all disattached from the primary structure by completely loosening the connections in this damage scenario. Again, 15 tests were conducted on the damaged structure. Acceleration data were collected and analyzed to effectively identify the damages. As shown in Figure 10, the reduction in the mean values of K3, K4, and K5 when compared with those of the original structure verified the effectiveness of the system ID-based damage detection method. Similar analysis with damage scenario no. 1 on the identified stiffness control chart can also present that the K2 and K6 are affected by the stiffness reduction at K3 to K5. And the reliability analysis clearly indicates the high risks of structural failure.

**Figure 11:**
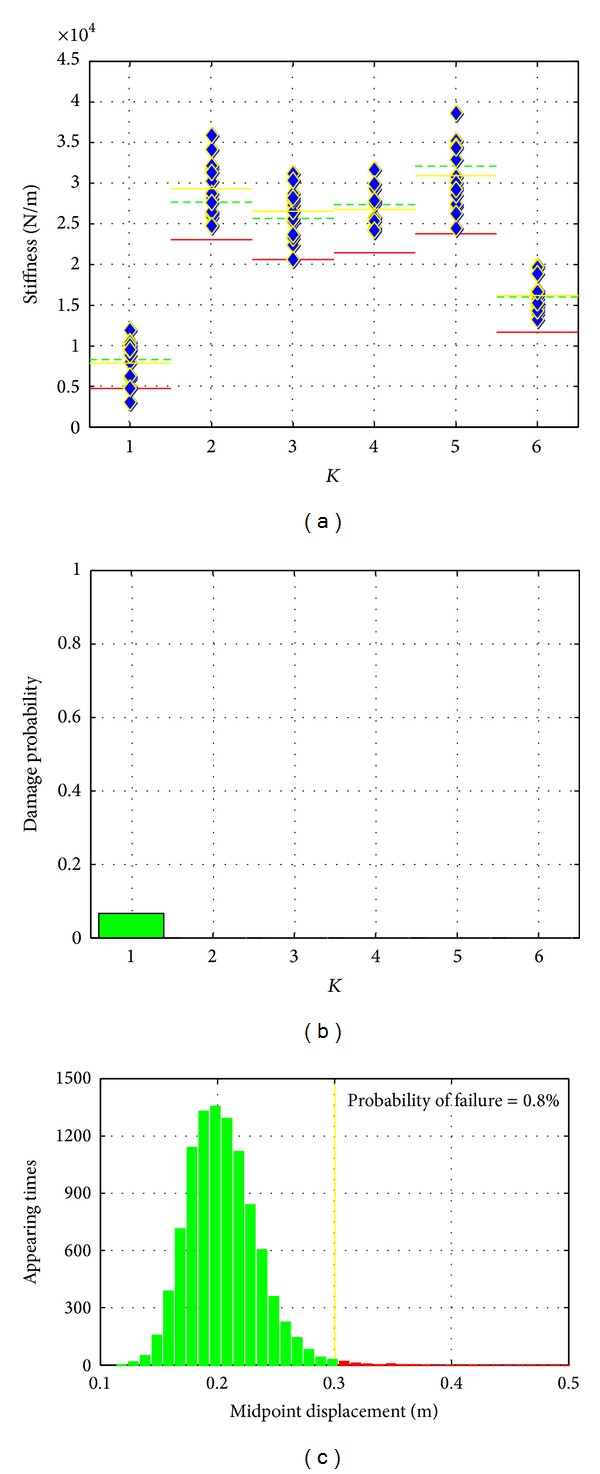
Results of damage scenario no. 4.

*Damage Scenario No. 4*. In this case, all disattached steel stripes were connected back to the primary structure by tightening the bolts at corresponding connections. In this way, a scenario which simulates the retrofitted structural system is created. The performances of the SHM method in this scenario are presented in Figure 11. The purely feature extraction and feature analysis results presented in Figures 11(a) and 11(b) show that the structure was restored to its original state and no stiffness reduction could be confidently classified. However, the system reliability analysis shows that the slight changes are still existing in the structure which increases the structural failure probability. Again, this verified that the system analysis step can provide more information which might not be possibly observed from purely pattern recognition procedure.

## 6. Conclusion

In this paper, the SHM problem is innovatively discussed on the context of KDD. By detailed comparison between SHM and KDD, a four-step SHM framework is proposed which expands SHM from pattern recognition problem to KDD problem. This framework emphasizes the importance of providing system working state-related knowledge to decision makers and incorporates both the structural system analysis methods and statistical KDD techniques in the individual step. Through clarifying the goal and hierarchy of extracting useful knowledge of SHM problems, the framework has potential to facilitate the further development of SHM. The experimental validation of the presented specific SHM method which combined the second-order structural parameter identification, statistical control chart analysis, and system reliability analysis shows the needs and advantages of this SHM framework on providing system knowledge and incorporating system identification method with statistical analysis tools.

## Figures and Tables

**Table 1:** Identified second-order parameters from well-controlled model testing stage.

Parameter	Test no. 1	Test no. 2	Test no. 3	Test no. 4	Test no. 5	Test no. 6	Average
*M*1 (Kg)	23	25	26	25	27	24	25
*M*2 (Kg)	30	30	25	31	24	31	29
*M*3 (Kg)	33	33	38	33	34	37	35
*M*4 (Kg)	30	30	25	31	26	31	29
*M*5 (Kg)	23	28	26	22	28	24	25
*K*1 (N/m)	7488	8813	7795	7992	8233	8938	8210
*K*2 (N/m)	25892	23752	21853	22004	26453	20696	23442
*K*3 (N/m)	28172	22252	21534	23398	22201	21243	23133
*K*4 (N/m)	28059	28442	25560	22136	24709	29241	26358
*K*5 (N/m)	25571	27927	25180	22984	22117	29703	25580
*K*6 (N/m)	13204	14813	13969	14946	13445	14123	14083
*D*1	0.04	0.04	0.05	0.04	0.04	0.04	0.04
*D*2	0.06	0.05	0.05	0.04	0.05	0.03	0.05
*D*3	0.06	0.04	0.06	0.05	0.04	0.04	0.05
*D*4	0.04	0.05	0.05	0.05	0.06	0.04	0.05
*D*5	0.04	0.03	0.07	0.06	0.06	0.04	0.05
